# Thymoma-associated myasthenia gravis coexisting with myotonic dystrophy: a case report

**DOI:** 10.1186/s40792-021-01223-6

**Published:** 2021-07-08

**Authors:** Michiyo Miyawaki, Mika Jikumaru, Kosuke Kamada, Noda Daiki, Miyuki Abe, Kentaro Anami, Hideya Takeuchi, Atsushi Osoegawa, Sintaro Iwao, Etsuro Matsubara, Kenji Sugio

**Affiliations:** 1 Department of Thoracic and Breast Surgery, Oita University Faculty of Medicine, 1-1 Idaigaoka Hasama-machi Yufu, Oita, 879-5593 Japan; 2 Department of Neurology, Oita University Faculty of Medicine, 1-1 Idaigaoka Hasama-machi Yufu, Oita, 879-5593 Japan

**Keywords:** Myotonic dystrophy, Thymoma, Myasthenia gravis

## Abstract

**Background:**

Myotonic dystrophy (dystrophia myotonica [DM]) is an autosomal-dominant inheritance, and myasthenia gravis (MG) is an autoimmune disease characterized by weakness of skeletal muscles. Cases of both DM and MG are extremely rare and distinguishing DM and MG symptoms is challenging.

**Case presentation:**

We herein report a 49-year-old woman presenting with subacute dyspnea and muscle weakness. She had previously been diagnosed with DM 24 years earlier. Computed tomography (CT) revealed an anterior mediastinal 32-mm solid mass that was suspected of being thymoma. The clinical features and neurological examination findings confirmed the diagnosis of thymoma-associated MG coexisting with DM. Intensive treatment for MG, including surgery, resulted in an improvement in some of her neurological symptoms.

**Conclusions:**

The symptoms of DM usually progress slowly, so the sudden exacerbation of symptoms indicates the involvement of other factors. It is important to be aware of these associations, as an early diagnosis with proper treatment will result in a better outcome.

## Background

Myotonic dystrophy (dystrophia myotonica [DM]) is an autosomal-dominant inheritance, and multisystem and heterogenous disease characterized by distal weakness, atrophy, and myotonia of skeletal muscles. Myasthenia gravis (MG) is an autoimmune disease characterized by weakness of skeletal muscles with diurnal variation. Distinguishing DM and MG symptoms is challenging. DM is associated with an increased risk of benign and malignant tumors. Although there have been some reports of cases of DM with thymoma, cases of DM accompanied by thymoma-associated MG are extremely rare.

## Case presentation

A 49-year-old woman was admitted to the hospital for the further examination of an anterior mediastinal tumor. She was suffering from weakness of the extremity muscles and dysphagia after catching a cold in the past month. She had been diagnosed with DM Type 1 (DM1) at 25 years by molecular genetic testing (CTG repeat expansion of DMPK gene proved by Southern blotting), and her older sister and niece had also been diagnosed as DM1, although she had not undergone a medical checkup. Before the instigating event, her symptoms of DM had been mild muscle weakness of the lower extremities and neck and face, myotonia of the hand, strabismus, and frontal balding.

**Fig. 1:**
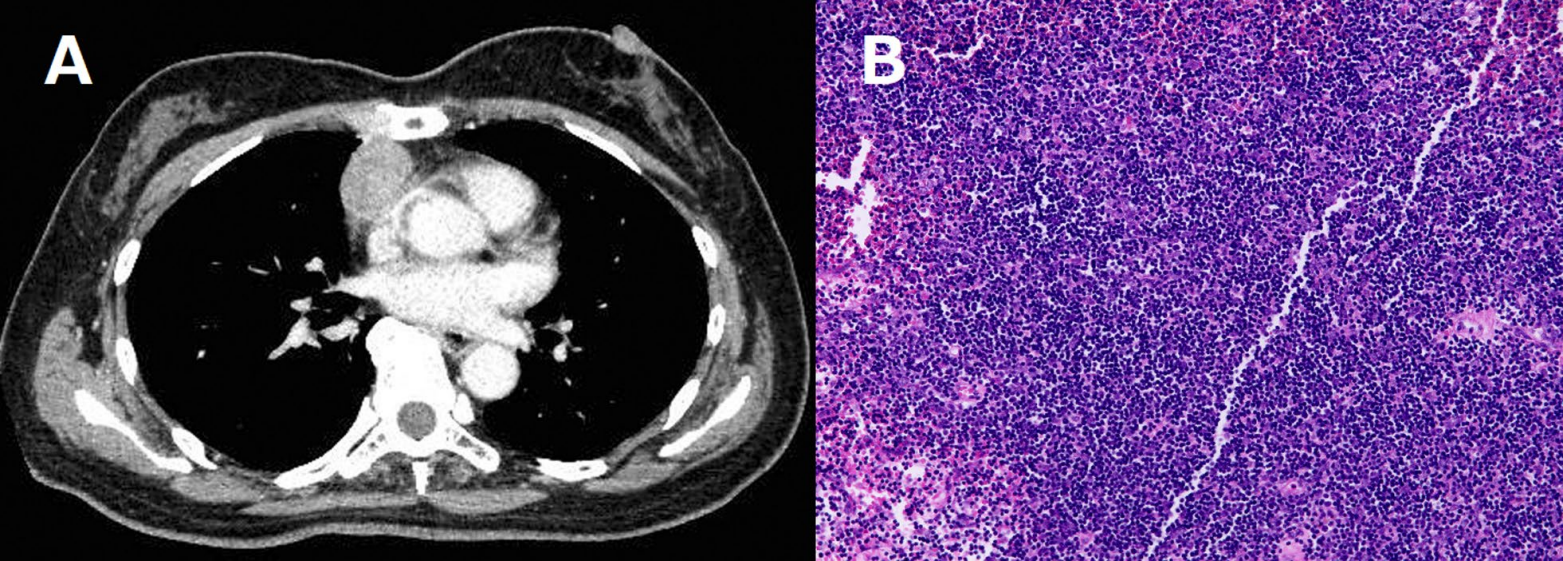
**a** Computed tomography findings of the chest showing a 32-mm solid tumor in the anterior mediastinum. **b** Hematoxylin–eosin staining of the tumor showing that the lymphocytic population obscures the epithelial cells (magnification × 200)

**Fig. 2:**
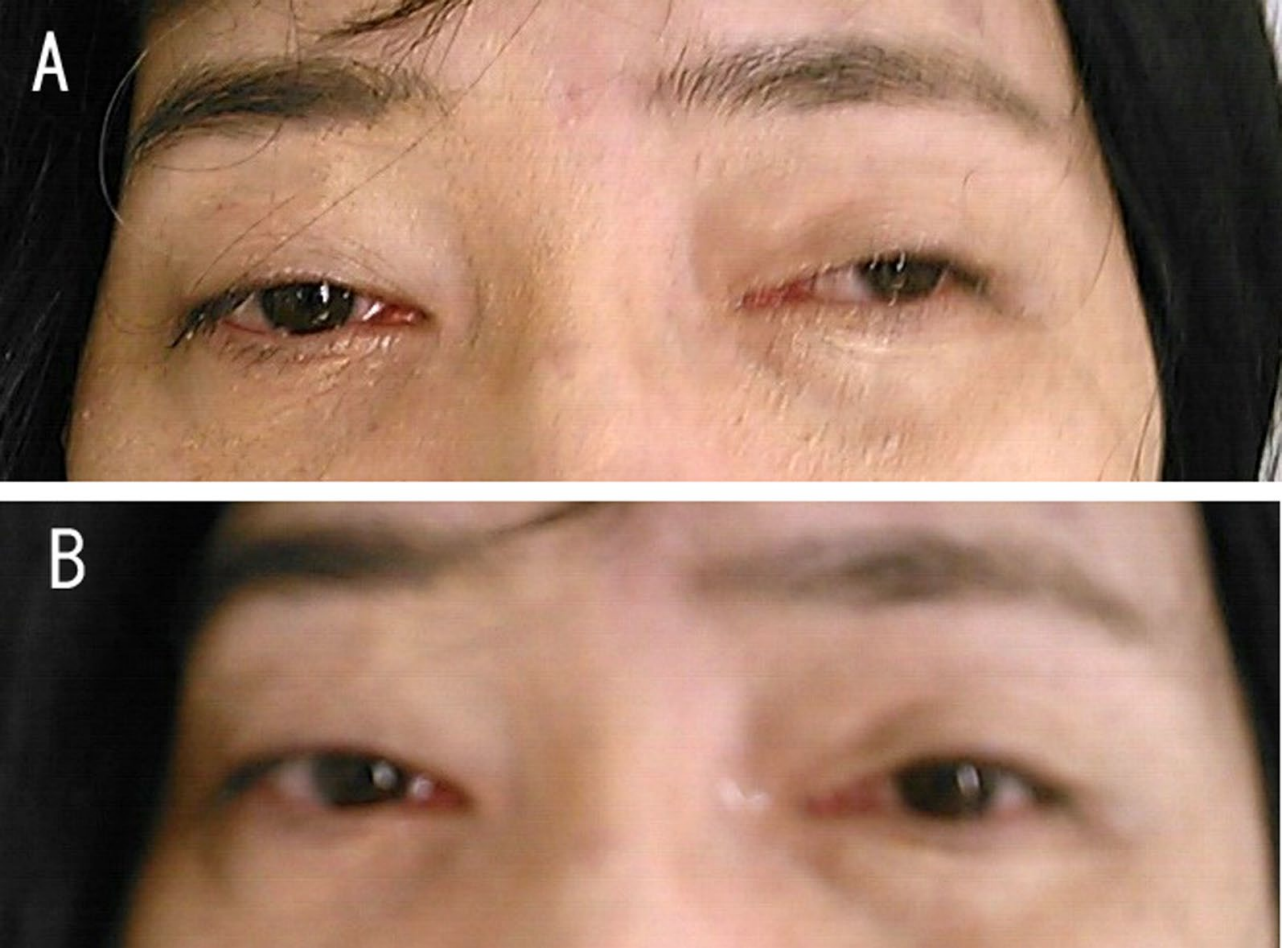
Patient has mild bilateral ptosis (**a**). The ptosis improved after the administration of edrophonium (**b**)

A neurological examination demonstrated ptosis and distal muscle weakness, gait disturbance, symptoms of bulbar paralysis, such as dysphasia, and dysarthria. Computed tomography (CT) revealed an anterior mediastinal 32-mm solid mass that was suspected of being thymoma (Fig. 1a). The acetylcholine receptor (AchR) antibody titer was 80 nmol/L (normal < 0.01 nmol/L). A tension test (edrophonium test) was positive (Fig. 2), and repetitive nerve stimulation tests with the median, accessary, and facial nerve showed a max value of 35% decremental response to 3–5-Hz repetitive stimulation. These findings indicated that she had MG (class IVb according to the Myasthenia Gravis Foundation of America [MGFA]). The clinical features and these examination findings confirmed the diagnosis of thymoma-associated MG coexisting with DM1.

**Fig. 3:**
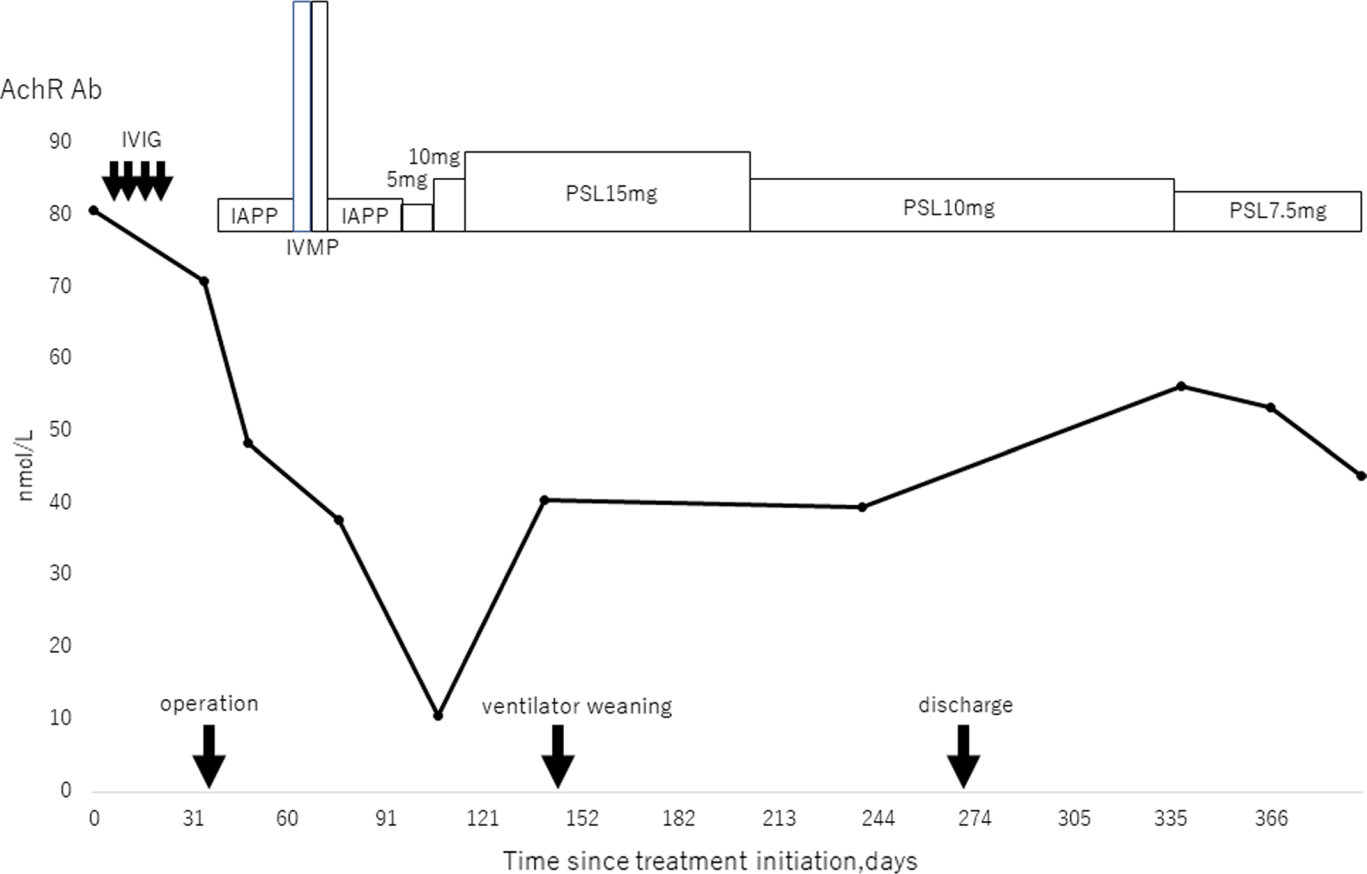
Course of treatment and changes in the acetylcholine receptor antibody titer. Immunity therapy reduced the value and slowly improved the respiratory failure. *IVIG* intravenous immunoglobulin, *IAPP* immunoadsorption plasma pheresis, *IVMP* intravenous methylprednisolone, *PSL* prednisolone

The blood counts and coagulation function test findings were within normal limits. An arterial blood gas analysis on room air revealed a pO_2_ of 55.4 mmHg and pCO_2_ of 60.1 mmHg. A respiratory function test showed a marked restrictive disorder pattern: %vital capacity (%VC) of 29.6% and forced expiratory volume 1.0 (s)% (FEV1%) of 84.5%. Weakness of the extremity muscles improved after pyridostigmine and intravenous immunoglobulin (IVIG). Nineteen days after admission, bilateral thoracoscopic extended thymothymectomy was performed. A pathological examination showed type B1 thymoma without invasion to capsule, and the tumor was diagnosed as pathological T1N0M0, stage I (Fig. 1b). Tracheotomy was performed the day after the operation because of postoperative crisis. Following this operation, two courses of intravenous methylprednisolone (IVMP) and two courses of immunoadsorption plasmapheresis (IAPP) were provided. Although the weakness of the extremity muscles improved gradually, the respiratory depression continued for a long time. After being weaned from the ventilator, oral intake was started 108 days after the operation, and she was transferred to a special facility for rehabilitation at 121 days after the operation. At 141 days after surgery, she was able to completely leave the ventilator. The AchR antibody level also decreased from a maximum of 80.8 to 10.6 following treatment for MG (Fig. 3). Respiratory failure and muscle weakness recovered to about the same degree as before the onset of MG, and she was discharged on day 270 after surgery.

## Discussion

Muscular dystrophy is a hereditary disease in which interstitial fibrosis and fatty degeneration progress under repeating cycles of skeletal muscle fiber degeneration and necrosis and incomplete regeneration. Among these diseases, DM is an autosomal-dominant-inherited non-coding repeat expansion disorder comprising two subtypes. DM1 is caused by unstable trinucleotide (CTG) repeat expansion in the 3′ untranslated region on the dystrophia myotonia-protein kinase (DMPK) gene, and type 2 DM (DM2) is caused by a tetranucleotide (CCTG) repeat expansion in intron 1 of the zinc finger 9 (ZNF9) gene [1]. DM1 is overwhelmingly more frequent, showing a general prevalence ranging from approximately 1:100,000 in some regions of Japan to 1:10,000 in Iceland; the global estimated prevalence of DM1 is reported to be 1:20,000 [2]. In contrast, only a few families with DM2 have reported in Japan.

Both DM and MG are neurological disorders with similar symptoms. DM is a multisystemic, heterogenous disease characterized by distal weakness, atrophy, and myotonia as well as symptoms of the heart, brain, gastrointestinal tract, endocrine, and respiratory systems. The diagnosis of DM is suspected in individuals with characteristic muscle weakness and confirmed by molecular genetic testing. However, no specific treatment exists for the progressive weakness. In contrast, MG is an autoimmune disease in which antibodies bind to acetylcholine receptors or to functionally related molecules in the postsynaptic membrane at the neuromuscular junction. The antibodies induce weakness of skeletal muscles, which is the only symptom of the disease. The weakness can be generalized or localized, is more proximal than distal, and nearly always includes eye muscles, with diplopia and ptosis. The diagnosis of MG is confirmed by the combination of relevant symptoms and electrodiagnostic tests, such as the repetitive nerve stimulation test and tensilon test (edrophonium test), as well as a positive test for specific autoantibodies, such as acetylcholine receptors. MG is a potentially reversible disorder with treatment options. DM shares clinical features that are similar in some respects to MG, including weakness and ptosis. As such, the coexistence of these two conditions, both of which are relatively uncommon, may be difficult to detect without a high level of suspicion and targeted laboratory testing. The present case was initially suspected to have exacerbated DM, but following a close examination, the onset of MG was proven.

Many reports have suggested that DM patients have an increased risk of developing benign and malignant tumors, such as cancers of the endometrium and cutaneous melanoma [3, 4], including thymoma [5]. There are several reports of DM associated with thymoma [5], but it is not clear whether this association is a syndrome or coincidence. Furthermore, cases of DM coexisting with thymoma-associated MG are very rare. An extensive literature review revealed only two published reports written in English [6, 7]. Both cases were diagnosed as MG by neurological tests and positive AchR antibody assays and were diagnosed as DM1 based on the family history and results of genetic testing. After an operation and radiation for thymoma, the MG-related neurological symptoms improved, as in the present case.

Special attention should be paid when administering general anesthesia to DM patients. In a retrospective analysis of 219 DM1 patients who underwent surgery under general anesthesia, most perioperative complications were related to the pulmonary system [8]. Delayed-onset apnea is most likely to develop in the first 24 h postoperatively. A decreased vital capacity pre-operatively serves as an important reminder that intensive-care unit specialists should be part of the team to evaluate the respiratory system and plan for appropriate post-operative care. Hypoxemia and hypercapnia are more likely to progress due to the lack of subjective symptoms characteristic of DM. She had received general anesthesia twice in the past, and the postoperative courses had been uneventful. This time, however, we paid close attention to her condition because of the coexistence of MG. Tracheotomy was performed the day after surgery under the presumption that long-term respiratory control would be required.

## Conclusions

The symptoms of DM usually progress slowly, so the sudden exacerbation of symptoms indicates the involvement of other factors. It is important to be aware of these associations, as an early diagnosis with proper treatment will result in a better outcome. In the present case, muscle weakness, dysphagia, and dyspnea progressed rapidly, and the symptoms improved following the treatment of MG.

## Data Availability

Not applicable.

## Abbreviations

DM: Myotonic dystrophy (dystrophia myotonica)
MG: Myasthenia gravis
